# Evaluation of microvascular changes in the perifoveal vascular network using optical coherence tomography angiography (OCTA) in type I diabetes mellitus: a large scale prospective trial

**DOI:** 10.1186/s12880-019-0391-8

**Published:** 2019-11-21

**Authors:** Javier Zarranz-Ventura, Marina Barraso, Anibal Alé-Chilet, Teresa Hernandez, Cristian Oliva, Jesus Gascón, Anna Sala-Puigdollers, Marc Figueras-Roca, Irene Vinagre, Emilio Ortega, Enric Esmatjes, Alfredo Adan

**Affiliations:** 1 Institut Clínic d’Oftalmología (ICOF), Hospital Clinic de Barcelona, C/ Sabino Arana 1, 08028 Barcelona, Spain; 2 Institut D’Investigacions Biomèdiques August Pi i Sunyer (IDIBAPS), Barcelona, Spain; 3 Diabetes Unit, Institut Clínic de Malalties Digestives i Metaboliques (ICMDM), Hospital Clinic de Barcelona, Barcelona, Spain

**Keywords:** Fovea, Macula, Diabetes mellitus, Type 1 Diabetes mellitus, Diabetic retinopathy, Vascular network, Optical coherence tomography angiography, OCTA

## Abstract

**Background:**

Diabetic retinopathy (DR) is the leading cause of blindness in type 1 Diabetes Mellitus (DM) patients, as a consequence of impaired blood flow in the retina. Optical coherence tomography angiography (OCTA) is a newly developed, non-invasive, retinal imaging technique that permits adequate delineation of the perifoveal vascular network. It allows the detection of paramacular areas of capillary non perfusion and/or enlargement of the foveal avascular zone (FAZ), representing an excellent tool for assessment of DR. The relationship of these microvascular changes with systemic factors such as metabolic control or duration of the disease still needs to be elucidated.

**Methods:**

Prospective, consecutive, large-scale OCTA study. A complete ocular examination including a comprehensive series of OCTA images of different scan sizes captured with 2 OCT devices (Cirrus HD-OCT, Carl Zeiss Meditec, Dublin, CA, USA, and Triton Deep Range Imaging OCT, Topcon Corp, Topcon, Japan) will be obtained as part of the yearly routine follow up visits in type 1 DM patients seen in the Diabetes Unit of the Endocrinology department which give written informed consent to participate in the project. The aim of this study is to investigate the relationship between OCTA-derived parameters and systemic factors, as metabolic control (Hb1Ac, lipid profile, cholesterol, etc), and other relevant clinical factors as demographics or duration of the disease.

**Discussion:**

This study is directed to investigate the relationship between the status of the perifoveal vascular network and systemic markers of the disease, and in particular to study whether these changes reflect those occurring elsewhere in the body affected by diabetic microvascular disease, as the kidneys or the brain. If these relationships were demonstrated, early detection of these microvascular changes by OCTA could lead to modifications in the pharmacological management of type 1 diabetic patients, as a way to reduce the risk of future complications in both the eye and other organs.

**Trial registration:**

ClinicalTrials.gov, trial number NCT03422965.

## Background

Diabetic retinopathy (DR) is the most important cause of blindness in type 1 DM patients [1, 2]. It has been postulated that selective loss of pericytes and thickening of the basement membrane in retinal capillaries occur as a result of exposure to elevated blood glucose over an extended period of time, damaging the retinal vessels and affecting the retinal blood flow in a time-dependant manner [3]. To evaluate the vascular flow in the retina of diabetic patients, fluorescein angiography (FA) requires an intravenous injection and has been relegated by the advent of non-invasive retinal imaging techniques [4–10] Optical coherence tomography (OCT) is a light-based technique that provides pseudo-histologic images of the retinal structure, which has become the main diagnostic tool in the management of retinal diseases [11]. Unfortunately, the information given by conventional OCT images is merely structural, and no information about blood flow or perfused areas can be obtained, being difficult to detect early changes in the retinal vascular network of diabetic patients.

Recently, a novel OCT-based technique called OCT angiography (OCTA) has been developed [12]. This OCTA technique is based in a newly developed software that employs an image processing algorithm, which analyses the decorrelation of the signal received by the OCT in a specific geographic location at two different timepoints. Similar to Doppler ultrasonography, the detection of two consecutive signals in a retinal vessel allows the detection of blood flow, and permits a two-dimensional reconstruction of the retinal vascular network. The main advantage of this technique is that it does not need the use of an intravenous dye, and therefore can be performed in a day-by-day basis in all patients. In the field of diabetic retinopathy, OCTA represents an interesting opportunity to evaluate the status of the perifoveal vascular network at different times of the disease, to identify early changes such as capillary dilation, enlargement of foveal avascular zone (FAZ), paramacular areas of capillary nonperfusion and presence of microaneurisms in a non-invasive way [13–18].

These preclinical alterations could be correlated with systemic factors such as time of diabetes evolution and metabolic control, and may also reflect those occurring elsewhere in the body affected by diabetic microvascular disease. Early detection of these changes could lead to modifications in the pharmacological management of patients to avoid microvascular complications in both retinal level and in other organs, such as the renal glomerulus.

The aim of this ongoing study is to evaluate the role of OCTA as a non-invasive screening tool for detecting early changes in the perifoveal vascular network in a large series of type 1 DM patients. The objectives of this project are to study the characteristics of the perifoveal capillary network using OCTA in patients with type 1 DM and controls, patients with type 1 DM with and without diabetic retinopathy, and patients with type 1 DM at different times of disease progression. Moreover, it is also directed to evaluate associations between the characteristics of the perifoveal capillary network with demographic factors, metabolic control and other risk factors in type 1 DM patients.

## Methods

### Study design

Cross sectional, exploratory study in a cohort of type I DM patients seen in the Endocrinology service in a 36-month period from May 2017, with prospective collection of OCTA images and relevant clinical data. This study is registered in the Clinical Trials website (ClinicalTrials.gov, trial number NCT03422965). The Standard Protocol Items Recommendations for Interventional Trials (SPIRIT) 2013 checklist was addressed in the present paper (Additional file 1).

### Ethics

This study was approved by the Institutional Review Board of Hospital Clinic of Barcelona (study protocol version 0.2, 23/11/2016). Written informed consent will be obtained for all participants.

### Study population

**Fig. 1:**
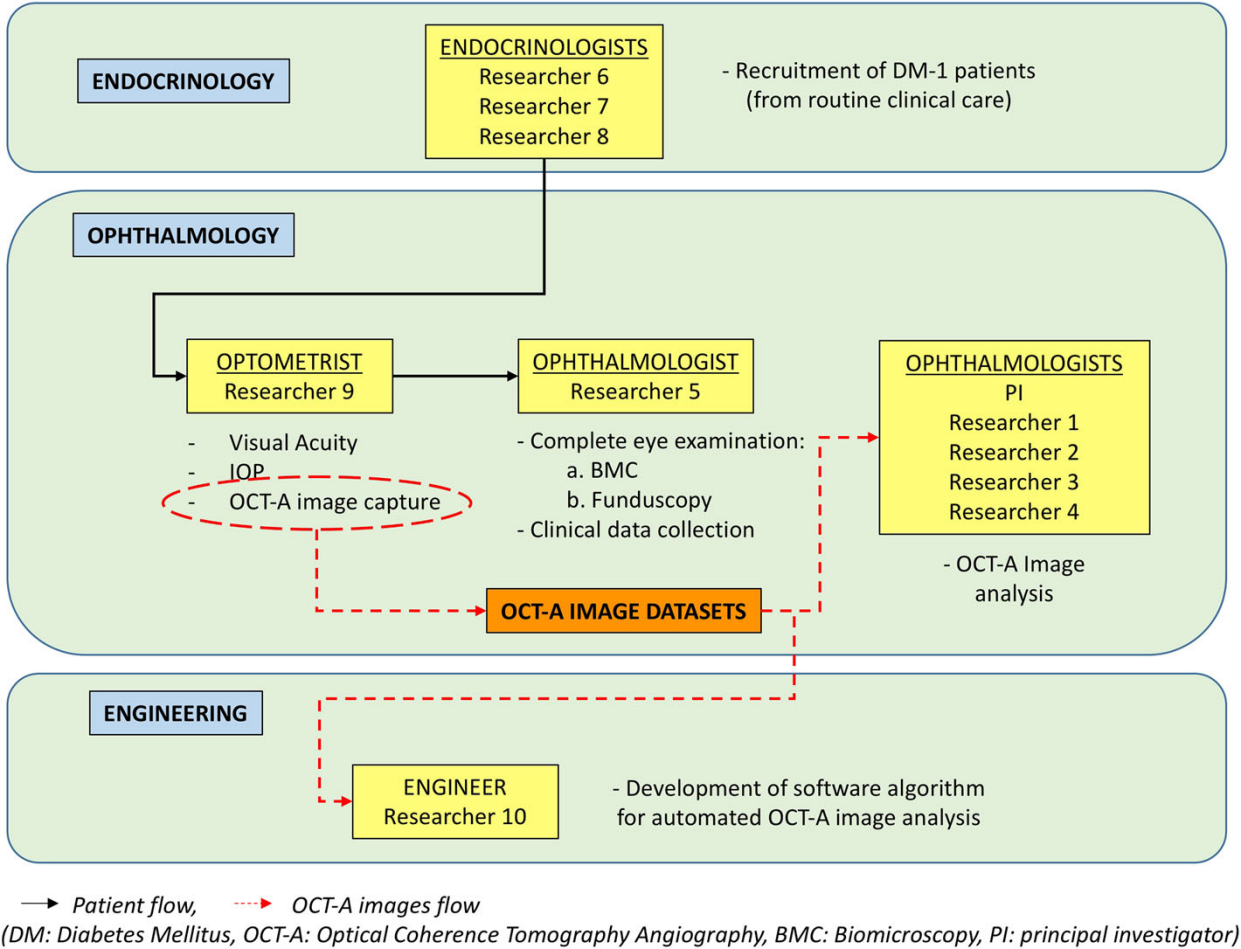
Patient flow diagram. Type 1DM patients seen in the Endocrinology service for their yearly revision will be invited to participate in the study (Researchers 6, 7 and 8). Those willing to participate will give informed consent and will be referred to the Ophthalmology service for a complete eye examination (Researcher 5) which include capture of a comprehensive battery of OCT and OCTA image datasets (Researcher 9). These images will be evaluated (Principal investigator and researchers 1–4) and processed with commercial and research softwares (Researcher 10) to obtain objective measurements of OCTA derived parameters (i.e. vessel density, etc.) which will be correlated to systemic markers (i.e. glycated haemoglobin, etc.)

Type I Diabetes Mellitus patients undergoing yearly follow up visits as per routine clinical care at the Diabetes Unit of the Endocrinology service of our center will be asked to participate in the study (Fig. 1).

### Sample size

Given that no normative databases are available for vessel density in normal eyes the project was designed in an exploratory basis. In our hospital, an estimate number of 2000 type I DM patients are seen in a yearly basis, which will be offered to enter the study voluntarily. All patients willing to participate during the study period will be included. We anticipate an estimated opt-out rate of 50%, with a final estimated sample size of 1000 patients.

### Inclusion and exclusion criteria

Patients with concomitant ocular pathology other than diabetic retinopathy detected in the ocular examination (uveitis, glaucoma, etc.) will be excluded from the analysis. Patients with inability to perform retinal images and ocular examinations (OCT, OCTA, fundus retinographies, biometry, etc.) or give written informed consent will not be included in the study.

### Study groups

Type I DM patients with and without existing diabetic retinopathy (DR) will be included in the study. Both groups will be classified by duration of diabetic disease (defined as date of type I DM diagnosis), in 3 main groups: A. ≤5 years from diagnosis, B. > 5 - < 15 years from diagnosis, and C. ≥15 years from diagnosis. Subgroup analysis will include also treatment type, glycemic control (glycaemia and HbA1c), body mass index, lipid profile and kidney function tests. A cohort of 100 normal eyes (controls) from a pool of healthy volunteers with clear media and without retinal disease will undergo retinal imaging with OCTA for comparison with type 1 DM cases images.

### Interventions

#### Systemic data

Systemic status of the diabetic disease will be evaluated in routine clinical care examinations in the Diabetes Unit of the Endocrinology service, and clinical data collected will include: Demographics, date of diagnosis, duration of disease, treatment type, glycaemia, HbA1c levels, concomitant pathology (hypertension, etc.), body mass index, lipid profile (total cholesterol, triglycerides, HDL-cholesterol, LDL-cholesterol) and kidney function tests (urinary albumin excretion, estimated filtration glomerular rate).

#### Ocular data

Patients identified in the Endocrinology service will be referred to the Ophthalmology service. In the eye clinics, a complete eye examination will be performed. Clinical data collected in this visit will include: Best-corrected visual acuity, biomicroscopy, intraocular pressure, retinal fundus exam and biometry (IOL Master, Carl Zeiss Meditec, Dublin, CA). A comprehensive battery of OCT and OCTA images will be performed, described below.

### OCTA images scanning protocols

Two optical coherence tomography angiography devices will be employed to obtain OCTA retinal images. Further retinal imaging techniques including fundus retinography (Optomap, Optos, Inc) and structural OCT will be performed.

#### Cirrus HD-OCT (Carl Zeiss Meditec, Dublin, CA, USA)

**Fig. 2:**
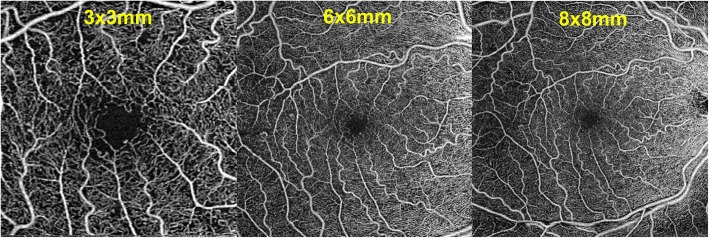
Examples of OCTA image datasets collected for each individual patient and eye included in the study (3 × 3, 6 × 6 and 8 × 8 mm scan sizes, Angioplex, Cirrus HD-OCT, Carl Zeiss Meditec, Dublin, CA, USA). Similar images were obtained with a different swept source device for each study eye (3 × 3, 6 × 6 and 9 × 9 mm, Triton Deep Range Imaging -DRI- OCT, Topcon Corp, Topcon, Japan)

A 6 × 6 mm OCT macular cube centered in the fovea will be captured, and measurements of retinal and choroidal thicknesses in each subfield of the Early Treatment Diabetic Retinopathy Study (ETDRS) grid will be obtained using the device’s manufacturer built-in software. Using the Angioplex OCTA, images will be captured with the 3 × 3, 6 × 6 and 8 × 8 mm scanning protocol (Fig. 2). This image will be subsequently loaded in the image processing software.

#### Triton deep range imaging (DRI) OCT (Topcon Corp, Topcon, Japan)

A 12 × 9 mm OCT macular cube centered in the fovea will be captured, and measurements of retinal and choroidal thicknesses in each subfield of the Early Treatment Diabetic Retinopathy Study (ETDRS) grid will be obtained using the device’s manufacturer built-in software. Using the OCTA scanning protocol, a 6 × 6 mm image of the scanned cube centered in the fovea will be obtained. This image will be subsequently loaded in the image processing software.

Controls: For validity of readings of the study device and as an internal normative database, a cohort of 100 normal eyes (controls) from a pool of healthy volunteers with clear media and without retinal disease will undergo retinal imaging and OCTA-derived measurements will be calculated. The mean intensity of the foveal avascular zone (FAZ) of these eyes will be determined to establish the reference for detection of flow in the macula.

### Data collection and data management

Systemic and ocular data will be obtained from the hospital electronic medical records system and will be collected in a pseudoanonymized database by the study team for confidentiality reasons. The OCT and OCTA images will be stored in the hospital servers and a back-up copy will be exported to encrypted hard disk drives that will remain physically in the hospital. All access to this data will be restricted only to the study team, and a formal data monitoring committee will not be constitued. Data sharing agreements will be specifically obtained from the sponsor of the study (Fundació Clínic para la Recerca Biomedica, at Hospital Clínic of Barcelona) by the study team for image analysis collaborations with third parties, as specifically indicated in the written informed consent signed by study participants.

### Main outcome measures

Main outcome measures to be determined will be A) parafoveal and perifoveal vessel density, B) total avascular area, and C) foveal avascular zone.

Comparisons will be made between these measurements in:

- controls and type I DM eyes,
- type I DM eyes with no DR and DR
- type I DM eyes with no DR and different duration of disease timeframes
- type I DM eyes with DR and different duration of disease timeframes

Secondary outcome measures include subgroup analysis of vessel density by treatment type, glycemic control (glycaemia and HbA1c), metabolic index, body mass index, and kidney function tests.

### Statistical methods

Descriptive and frequency statistics will be used to assess qualitative variables. Normality of quantitative variables (i.e. pixel area) will be examined using histograms and the Kolmogorov-Smirnov test protected by the Bonferroni correction. To assess differences in quantitative measurements between study groups, parametric and non-parametric tests will be performed as appropriate, respectively. Correlation between clinical data and OCTA derived measurements will be analyzed using a multivariate regression model to assess the effects of clinical characteristics on these values. Visual acuity measured in Snellen notation was converted to logMAR (logarithm of the minimum angle of resolution) equivalents for the purposes of statistical analysis. VA values recorded as counting fingers (CF), hand movements (HM), and perception of light (PL) were converted to 2.1, 2.4, and 2.7 LogMAR, respectively. A *p* value of less than 0.05 will be considered statistically significant. All statistical analysis will be performed using IBM SPSS Statistics software version 21.0 (IBM Corp., Armonk, NY, USA).

## Discussion

This study is directed to evaluate the potential role of OCTA as a useful tool to inform the status of the macular microvascular network in type 1 Diabetes Mellitus patients. Its prospective nature and collaborative design between endocrinologists and ophthalmologists may allow the recruitment of a large cohort of type 1 DM patients and controls to permit the study of potential relationships between the perifoveal vascular network parameters obtained with OCTA and systemic markers of the disease. The positive or negative results of this study will be communicated in scientific meetings and publications. If these relationships are identified OCTA may be included in the screening program of type 1 DM patients, as these changes may preceed the progression to more advanced stages of diabetic retinopathy (i.e. from non DR to mild DR stages), as well as reflect the status of the microvasculature in other parts of the body only accessible by invasive procedures, such as kidney or heart biopsies. The potential of this technology will be evaluated in this and future trials.

## Supplementary information

**Additional file 1.** Standard protocol items: recommendations for interventional trials (SPIRIT) 2013 checklist for the current study.

## Data Availability

The datasets used and/or analysed during the current study are available from the corresponding author on reasonable request.

## Abbreviations

DR: Diabetes Mellitus
DR: Diabetic retinopathy
ETDRS: Early Treatment Diabetic Retinopathy Study
FAZ: Foveal Avascular Zone
OCT: Optical Coherence Tomography
OCTA: Optical Coherence Tomography Angiography
